# Anidulafungin and Micafungin Concentrations in Cerebrospinal Fluid and in Cerebral Cortex

**DOI:** 10.1128/AAC.00275-20

**Published:** 2020-06-23

**Authors:** Jana Marx, René Welte, Tiziana Gasperetti, Patrizia Moser, Ronny Beer, Martin Ortler, Martina Jeske, Ramona Stern, Andreas Pomaroli, Michael Joannidis, Romuald Bellmann

**Affiliations:** a Clinical Pharmacokinetics Unit, Division of Intensive Care and Emergency Medicine, Department of Internal Medicine I, Medical University of Innsbruck, Innsbruck, Austria; b Department of Pathology, Medical University of Innsbruck, Innsbruck, Austria; c Neurological ICU, Department of Neurology, Medical University of Innsbruck, Innsbruck, Austria; d Neurosurgical ICU, Department of Neurosurgery, Medical University of Innsbruck, Innsbruck, Austria; e Hospital Pharmacy, Innsbruck General Hospital, Innsbruck, Austria; f Transplant ICU, Department of Anesthesia and Critical Care, Centre of Operative Medicine, Innsbruck General Hospital and Medical University of Innsbruck, Innsbruck, Austria; g Division of Intensive Care and Emergency Medicine, Department Internal Medicine I, Medical University of Innsbruck, Innsbruck, Austria

**Keywords:** echinocandins, antifungal target-site pharmacokinetics, CNS penetration, fungal meningoencephalitis, CNS candidiasis, critically ill

## Abstract

Anidulafungin and micafungin were quantified in cerebrospinal fluid (CSF) of critically ill adults and in cerebral cortex of deceased patients. In CSF, anidulafungin levels (<0.01 to 0.66 μg/ml) and micafungin levels (<0.01 to 0.16 μg/ml) were lower than those in plasma concentrations (0.77 to 5.07 and 1.21 to 8.70 μg/ml, respectively) drawn simultaneously. In cerebral cortex, anidulafungin and micafungin levels were 0.21 to 2.34 and 0.18 to 2.88 μg/g, respectively. Thus, MIC values of several pathogenic *Candida* strains exceed concentrations in CSF and in brain.

## Abstract (precis)

Anidulafungin and micafungin were quantified in cerebrospinal fluid (CSF) of critically ill adults and in cerebral cortex of deceased patients. In CSF, anidulafungin levels (<0.01 to 0.66 μg/ml) and micafungin levels (<0.01 to 0.16 μg/ml) were lower than those in plasma concentrations (0.77 to 5.07 and 1.21 to 8.70 μg/ml, respectively) drawn simultaneously. In cerebral cortex, anidulafungin and micafungin levels were 0.21 to 2.34 and 0.18 to 2.88 μg/g, respectively.

## TEXT

The echinocandins anidulafungin and micafungin are recommended for treatment of invasive candidiasis (1, 2). Candidiasis of the central nervous system (CNS) is associated with a high mortality of 80% to 100% in immunocompromised patients (3, 4). Because knowledge on penetration of echinocandins into human CNS is limited, we quantified anidulafungin and micafungin in the cerebrospinal fluid (CSF) of critically ill patients and the cerebral cortex of deceased patients.

The study was approved by the local ethics committee and performed in accordance with the Declaration of Helsinki and Austrian law. Written informed consent for scientific use of CSF and blood samples was granted by competent patients. *Post hoc* consent was obtained from patients who were incompetent at the time of enrollment. Autopsy samples were taken from deceased patients who, on admission, had permitted scientific use of residual specimens taken for clinical laboratory tests.

CSF was taken during diagnostic lumbar puncture (LP) or via external ventricular drain (EVD) from critically ill adults treated with anidulafungin or micafungin. Simultaneously, we took 2-ml arterial blood samples. CSF and plasma were stored at –80°C.

Anidulafungin and micafungin concentrations were quantified by high-performance liquid chromatography and UV detection (HPLC-UV) as described previously (5). Noncompartmental pharmacokinetics was calculated with Kinetica 2000 (InnaPhase Corporation, Champs-sur-Marne, France). The area under the concentration-time curve from 0 to 24 h (AUC_0–24_) was computed with the log-linear method when the concentration in a trapezoid decreased or with the trapezoidal method when the concentration increased.

The cerebral cortex of deceased patients who had received anidulafungin or micafungin within their last 30 days of life was sampled during autopsy, which is routinely performed for quality assurance in Austrian hospitals. Anidulafungin and micafungin were extracted from 0.2 g of tissue by addition of 250 μl of acetonitrile and 250 μl of methanol (Sigma-Aldrich, Vienna, Austria), homogenization with a Precellys homogenizer (Bertin Instruments, Montigny-le-Bretonneux, France) at 4,500 rpm (twice for 25 s, 2-s break), and centrifugation for 5 min at 8°C and 4,665 × *g*. For calibration, we used porcine brain samples spiked with anidulafungin or micafungin and homogenized and proceeded in the same manner. Both echinocandins were quantified by HPLC-UV at 306 nm (5). The lower limit of quantification was 0.05 μg/g for anidulafungin and 0.10 μg/g for micafungin. The extraction recovery from brain was ∼40% and ∼60% for anidulafungin and micafungin, respectively.

The significance of the difference between CSF and plasma concentrations was assessed by Wilcoxon matched-pairs test, and the difference between the penetration ratio (PR) of anidulafungin and PR of micafungin was calculated by Mann-Whitney *U* test using IBM SPSS statistics 24.0. PR was the ratio between the AUC_0–24_ in CSF and that in plasma over 24 h (AUC_0–24 CSF_/AUC_0–24 plasma_), when multiple samples were drawn via EVD. For single samples drawn by LP, PR was the ratio between the concentration in CSF and that in plasma (C_CSF_/C_plasma_).

**FIG 1:**
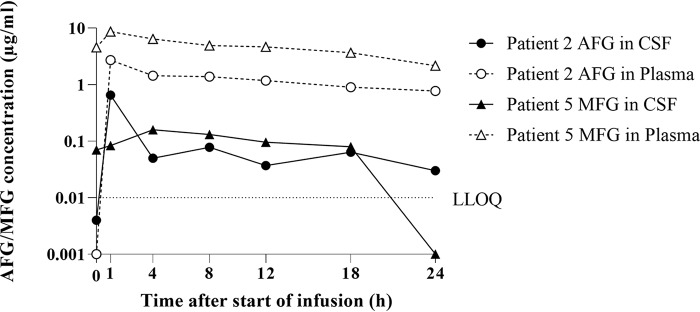
Concentration-time profiles of anidulafungin (AFG) and micafungin (MFG) in cerebrospinal fluid (CSF) and plasma over the dosage interval of 24 h.

CSF samples were obtained from three patients on anidulafungin and three patients on micafungin. One patient on anidulafungin (patient 2) and one patient on micafungin (patient 5) had undergone EVD (Table 1; Fig. 1). CSF concentrations of anidulafungin (<0.7 μg/ml) and micafungin (<0.2 μg/ml) were lower than the corresponding plasma concentrations (*P* < 0.05 and *P* < 0.01, respectively) (Table 1; Fig. 1). PRs of anidulafungin and micafungin were similar (*P* = 0.40). In the cerebral cortex of four deceased patients, anidulafungin had reached concentrations of 0.21 to 2.34 μg/g. Brain concentrations of micafungin were <0.10 to 2.88 μg/g (*n* = 6). Anidulafungin and micafungin were measurable even 13 and 10 days, respectively, after treatment (Table 2).

**TABLE 1:** Penetration of anidulafungin or micafungin into cerebrospinal fluid of critically ill patients

Sample source and patient no.^*a*^	Main diagnosis^*b*^	Age (yr)	Sex	Wt (kg)	Drug^*c*^	Cumulative dose (mg)	Treatment day^*d*^	CSF concn (μg/ml)	Plasma concn (μg/ml)	PR^*e*^	Time from infusion (h)^*f*^	Sample type	*C*_max_ (μg/ml)^*g*^	*C*_min_ (μg/ml)^*h*^	AUC_0–24_ (μg · h/ml)	*T*_max_ (h)^*i*^	*t*_1/2_ (h)^*j*^
Lumbar puncture																	
1	ALL relapse, st. p. HSCT, pneumonia	25	F	63	AFG	300	3	0.05	3.83	0.01	3.0						
3	C. krusei, peritonitis, candidemia, st. p. LTX	40	F	46	AFG	2,500	24	<0.01	5.07	<0.002	0.5						
4	Tick-borne encephalitis, pneumonia, sepsis	83	M	75	MFG	200	2	0.09	1.21	0.08	16.0						
6	NK-T-cell lymphoma, sepsis	48	M	79	MFG	1,300	13	0.10	3.51	0.03	2.5						
External ventricular drainage																	
2	SAH, C. albicans meningitis,^*k*^ candidemia	56	M	160	AFG	200	1			0.07		CSF	0.66	0.03	2.09	1	7.90
												Plasma	2.72	0.77	29.41	1	14.70
5	ICH, CAA, sepsis	72	F	50	MFG	200	2			0.02		CSF	0.16	<0.01	2.01	4	13.80
												Plasma	8.70	2.16	112.70	1	12.90

a When cerebrospinal fluid (CSF) was sampled via external ventricular drainage (EVD), the collection bags were changed before and 1, 4, 8, 12, 18, and 24 h after start of infusion. Simultaneously with LP or with the changes of the collection bags, 2-ml blood samples were taken from an arterial line using heparinized vials (Sarstedt, Nümbrecht, Germany). Lower limit of quantification is 0.01 μg/ml for AFG and MFG; steady state had not yet been reached in patients 1, 2, 4, and 5.

b ALL, acute lymphocytic leukemia; st. p., status post; HSCT, hematopoietic stem cell transplantation; LTX, liver transplantation; SAH, subarachnoid hemorrhage; ICH, intracerebral hemorrhage; CAA, cerebral amyloid angiopathy.

c AFG, anidulafungin; MFG, micafungin.

d Day of echinocandin treatment; anidulafungin (Ecalta, Pfizer Limited, Sandwich, Kent, UK) and micafungin (Mycamine, Astellas, Tokyo, Japan) were administered for suspected or proven invasive candidiasis at the discretion of the treating physician.

e Penetration ratio, ratio between AUC_0–24_ in CSF and in plasma.

f Time between start of the anidulafungin or micafungin infusion and sampling.

g Peak concentration.

h Trough concentration.

i Time to *C*_ma.x_.

j Half-life.

k A *Candida* meningoencephalitis was diagnosed 2 days after sampling and required a switch to liposomal amphotericin B and flucytosine.

**TABLE 2:** Anidulafungin and micafungin concentrations in autopsy samples of cerebral cortex

Patient no.	Main diagnosis^*a*^	Age (yr)	Sex	Wt (kg)	Drug	Cumulative dose (mg)	Treatment duration (days)^*b*^	Interval between last AFG/MFG administration and death (h)	Interval between death and sampling (h)^*c*^	Concn (μg/g)
7	COPD, pneumonia	74	F	135	AFG	700	6	32	14	0.21
8	DLBCL, septic shock	68	F	85	AFG	1,800	17	313	25	0.28
9	DLBCL, st. p. HSCT, ileus, pneumonia	45	F	43	AFG	1,500	14	12	15	2.34
10	Sepsis, peritonitis after sigmoid perforation	70	F	50	AFG	1,200	11	29	21	1.58
11	Burkitt-lymphoma relapse, st. p. HSCT	38	M	80	MFG	1,100	19	712	58	<0.10
12	Wound infection (C. albicans), septic shock, osteomyelofibrosis	60	M	92	MFG	5,300	28	230	85	1.52
13	Cholangiocarcinoma, biliary-pleural fistula, sepsis	49	M	80	MFG	1,800	18	111	38	0.18
14	St. p. LuTX, ischemic stroke, pneumonia	58	M	60	MFG	500	4	25	74	2.88
15	Fungal endophthalmitis, pneumonia, COPD	76	M	81	MFG	300	3	324	80	<0.10
16	St. p. LTX, hepatic artery occlusion, wound infection	71	F	85	MFG	4,000	39	235	29	0.19

a COPD, chronic obstructive pulmonary disease; DLBCL, diffuse large B-cell lymphoma; st. p., status post; HSCT, hematopoietic stem cell transplantation; LuTX, lung transplantation.

b Treatment duration, days of echinocandin therapy.

c Patients had deceased during or within 30 days after treatment with anidulafungin (AFG) or micafungin (MFG). AFG (Ecalta, Pfizer Limited, Sandwich, Kent, UK) and MFG (Mycamine, Astellas, Tokyo, Japan) were stable in brain tissue for at least 96 h at 4°C, which was the storage temperature of the corpses. AFG and MFG had been administered for suspected or proven invasive candidiasis at the discretion of the treating physician. The lower limit of quantification is 0.05 μg/g for AFG and 0.10 μg/g for MFG.

In some of the CSF and brain specimens, measured anidulafungin and micafungin concentrations were below the MICs reported for relevant *Candida* species (i.e., 0.008 to 4.0 μg/ml) (6). In the CSF of patients 2 and 5, the target AUC_0–24_/MIC ratios of 2,782 and 5,299 suggested for anidulafungin and micafungin, respectively, have not been achieved, not even for highly susceptible *Candida* strains (6, 7). However, the relevance of *in vitro* MIC values for antifungal activity of echinocandins in CSF and in CNS remains to be clarified. In CSF, protein binding of anidulafungin and micafungin is unknown. *In vitro*, protein binding obviously affects MICs (8, 9). We did not separate protein-bound from free echinocandins. In our small and heterogeneous study population, only two patients (patients 3 and 6) reached steady state, when CSF was taken by LP, and only two patients (patients 2 and 4) suffered from CNS infections that might have enhanced permeability of the blood-brain barrier (10). However, CSF concentrations and PRs of anidulafungin and micafungin in patients 2 and 4 were similar to those in our other patients.

During cerebral aspergillosis, the 2-fold maximum dose of micafungin (i.e., 300 mg/day) achieved CSF concentrations of <0.02 μg/ml (11). After intracranial hemorrhage, micafungin CSF levels amounted to 0.019 to 4.66 μg/ml (12). Caspofungin was undetectable in CSF during meningeal coccidioidomycosis and in 9 of 11 CSF samples from children with hematological malignancies (13, 14). In infants with meningitis, daily high doses of 8 to 10 mg/kg of micafungin resulted in CSF concentrations of 0.80 to 1.80 μg/ml (15). In neonatal rats, anidulafungin brain concentrations of 1.60 and 4.40 μg/g were measured (16). Along with the higher dosage, the immaturity of the blood-brain barrier might explain the higher penetration into CSF and brain.

In brain specimens, we cannot rule out minor agonal or postmortem changes of anidulafungin and micafungin concentrations, although anidulafungin and micafungin were stable in the brains of deceased patients for at least 96 h. Brain specimens consist of various compartments, e.g., different cells, extracellular matrix, and blood vessels. Anidulafungin and micafungin were not quantified on a cellular level or in different brain areas. Echinocandin extraction from human brain might yield slightly lower recovery than extraction from external standards. In rats, [^3^H]caspofungin brain concentrations were ≤0.16 μg eq/g (17). In rabbits, micafungin brain concentrations were <0.19 μg/g, and those of anidulafungin were <4.0 μg/g (18, 19). In a biopsy sample of a brain abscess, a micafungin concentration of 0.26 μg/g was measured (20).

In conclusion, anidulafungin and micafungin concentrations in CSF and in brain specimens were below the *in vitro* MIC values of several pathogenic *Candida* strains. Studies on target-site pharmacodynamics are required for assessment of antifungal efficacy.

(Parts of the data were presented at the 23rd and 25th Scientific Symposium of the Austrian Pharmacological Society, 2017 and 2019, Innsbruck, Austria.)
